# SOCIAL NETWORK TYPES TRANSITIONS, HEALTH AND WELL-BEING-A PERSON-CENTERED APPROACH AMONG CHINESE OLDER ADULTS

**DOI:** 10.1093/geroni/igae098.4283

**Published:** 2024-12-31

**Authors:** Shuting Dai

**Affiliations:** City University of Hong Kong, Hong Kong, China (People’s Republic)

## Abstract

The social networks of older individuals reﬂect personal life history and cultural factors. However, little is known about stability and change in social network types over time in China. The purposes of this study were to (1) investigate transitions in social network types of Chinese older adults; and (2) examine the transitions of the relationship of the social network types to the measures of health and well-being. Data were drawn from ﬁve waves of the China Health and Retirement Longitudinal Study to examine the changes in social network heterogeneity in old Chinese cohort. Data from 19395 observations of 7321 older adults (65 years and older) were considered. Latent transition analysis (LTA) was conducted using seven social network-related variables. The identified typologies were then regressed on the indicators of health and well-being. We found 5 social network types at both waves, representing the most to the least diverse types–diverse, unmarried and diverse, extended family, immediate family, and restricted. About 68% of respondents between waves retained their social network type, whereas 19% transitioned into more diverse types and 13% into less diverse types. Compared with the restricted network, older adults in all other networks were more likely to report higher self-rated health, higher life satisfaction, and lower depressive symptoms at both waves. The findings capture the dynamics in social network composition among older adults in China. A person-centered approach is recommended for policymakers to understand social network transitions, and promoting social network diversity in later life may contribute to healthy aging.

